# A Formal Representation of the WHO and UNICEF Estimates of National Immunization Coverage: A Computational Logic Approach

**DOI:** 10.1371/journal.pone.0047806

**Published:** 2012-10-25

**Authors:** Anthony Burton, Robert Kowalski, Marta Gacic-Dobo, Rouslan Karimov, David Brown

**Affiliations:** 1 Department of Immunization, Vaccines and Biologicals, World Health Organization, Geneva, Switzerland; 2 Department of Computing, Imperial College London, London, United Kingdom; 3 Division of Policy and Practice, United Nations Children's Fund, New York, New York, United States of America; Tulane University School of Public Health and Tropical Medicine, United States of America

## Abstract

Production of official statistics frequently requires expert judgement to evaluate and reconcile data of unknown and varying quality from multiple and potentially conflicting sources. Moreover, exceptional events may be difficult to incorporate in modelled estimates. Computational logic provides a methodology and tools for incorporating analyst's judgement, integrating multiple data sources and modelling methods, ensuring transparency and replicability, and making documentation computationally accessible. Representations using computational logic can be implemented in a variety of computer-based languages for automated production. Computational logic complements standard mathematical and statistical techniques and extends the flexibility of mathematical and statistical modelling. A basic overview of computational logic is presented and its application to official statistics is illustrated with the WHO & UNICEF estimates of national immunization coverage.

## Introduction

Official statistics, particularly at the international level [1]–[4] often rely on potentially conflicting data of unknown and varying quality from multiple sources. Expert judgement is frequently required to evaluate and reconcile these data and shocks to the system - perturbations in the system environment, such as civil unrest, that result in unpredicted system behaviour - and deviation from general trends and patterns may be difficult to incorporate in conventional mathematical or statistical models. Current methods for ensuring transparency, replicability, and sufficiently detailed documentation in these circumstances are challenging, cumbersome, and frequently inadequate.

Computational logic [5], [6], a form of symbolic logic developed in computer science and artificial intelligence, provides a powerful and flexible methodology and set of tools that is especially well-suited for formally describing complex situations. Models described in computational logic can also take advantages of computer-based languages for large scale implementation.

Since 2000 the World Health Organization (WHO) and the United Nations Children's Fund (UNICEF) have made annual estimates of national infant immunization coverage for selected vaccines [7]. A principal use of these estimates is to monitor international goals [8]; for example, measles immunization coverage is an indicator for tracking progress towards the Millennium Development Goal 4, Reduction of Child Mortality [9].

Estimates are based on reports to WHO and UNICEF submitted by national authorities and are supplemented with results from nationally representative household or community surveys. Local staff, primarily national immunization system managers and WHO/UNICEF regional and national staff, are consulted for information on the performance of specific immunization systems and factors that might influence or bias empirical data. WHO and UNICEF estimates are derived through a country-by-country review of available data informed and constrained by a set of heuristics - some of which are described below - and make only limited use of statistical and mathematical models. While the final estimates may not differ from data reported by national authorities, they constitute an independent technical assessment by WHO and UNICEF of the national immunization system performance. Annual country-specific estimates from 1980 are available at: http://www.who.int/immunization_monitoring/en/globalsummary/wucoveragecountrylist.cfm and http://www.childinfo.org/immunization_countryreports.html.

The informal articulation and manual application of the estimation procedure has led, in some instances, to estimates that are inconsistent (that is, do not adhere to the appropriate heuristics), irreproducible results and to insufficiently informative accompanying documentation. To address these issues and improve the transparency of the methods, computational logic has been applied to formally represent rules, data and decisions from which the WHO and UNICEF estimates of national immunization coverage (WUENIC) may be logically inferred.

The declarative nature of the formalization lends itself to a fairly direct translation to logic programming and expert-system computer languages. To take advantage of automated data processing, the formal representation has been implemented in the general-purpose logic programming language Prolog [10], which implements a version of computational logic. The implementation was first used in May 2010 to support the production of estimates for the period 1997–2009. It has been used subsequently in 2011 and 2012 to extend the estimates to the periods 1997–2010 and 1997–2011 respectively. The formal representation and Prolog code are available at: http://www.sites.google.com/site/wuenic/.

## Methods

Computational logic, which is both simpler and more powerful than conventional symbolic logic, is used to represent knowledge and to derive logical consequences of that knowledge. Knowledge represented in computational logic can be viewed as a relational database extended by rules expressed in logical form. Such representations are often called “knowledge bases”.

In computational logic, logical consequences of information in a knowledge base are derived by means of inference rules, which implement a mechanical reasoning procedure. In our application, domain-specific knowledge of immunization coverage is represented in computational logic and the inference rules are used to derive estimates of immunization coverage. The domain-specific knowledge consists of:

1. *data* and other domain-specific information relevant to immunization coverage. The data include coverage reported by national authorities and results from national household or community surveys. Other information includes knowledge about the quality and relevance of reported data and surveys (e.g. survey sample size), assessments of national monitoring systems and the occurrence of programmatic and exogenous factors influencing immunization system performance (e.g., vaccine supply shortages, changes in immunization policies, civil unrest);
2. *rules* representing the heuristics and methods used to derive estimates from the data and information, to define domain-specific concepts, and perform computations.
3. *decisions* made by the working group both to override and to augment the rules. Such decisions are explicitly identified and are accompanied with a documented explanation.

The data, rules and decisions are represented in computational logic by means of two simple kinds of sentences: *atomic sentences* (also called *facts*), which have no subparts that are also sentences, and *conditionals*, which have the form ***if*** *condition(s)* ***then*** *conclusion* or equivalently, *conclusion* ***if*** *condition(s)*. Such conditionals (also called *implications*) combine an atomic *conclusion* with a conjunction of *conditions* [5].

In the remainder of this section, the logic-based approach is presented and illustrated with simplified examples taken from our application. See the Annotated Bibliography S1: Knowledge representation and reasoning using computational logic: an annotated bibliography for additional material on knowledge representation and reasoning.

### Facts

*An atomic sentence (*or *fact)* consists of a predicate (or relationship) with a number of arguments (or parameters). In symbolic notation, facts are written with the predicate first, followed by the arguments, separated by commas and surrounded by parentheses. For example, *data reported by national authorities* is represented as:

reported (*country, vaccine, year, coverage*)

where reported is a predicate and *country, vaccine, year, coverage* are the arguments of the predicate. *coverage* represents the proportion of children below one year of age in the *country* vaccinated during the *year*with the *vaccine*, as reported by the national authorities.

For example, the fact that coverage for the third dose of diphtheria, tetanus and pertussis vaccine (DTP3) in 2004 reported by the Egyptian national authorities was 97% is represented as:

reported (egy, dtp3, 2004, 97)

*Survey results* are represented in the form:

survey (*country, vaccine, year, coverage*)

For example, the fact that a Demographic and Health Survey (DHS) found 93.5% DTP3 coverage in Egypt for a sample of children born in 2004 is represented as:

survey (egy, dtp3, 2004, 93.5)

In relational databases, predicates are relations, which can be viewed as tables. For example, the reported and survey predicates could be pictured as in tables 1 and 2:

**Table 1:** Reported data.

*Country*	*Vaccine*	*Year*	*Coverage*
egy	dtp3	2004	97
egy	dtp3	2005	96
…….	….	….	…….

**Table 2:** Survey data.

*Country*	*Vaccine*	*Year*	*Coverage*
egy	dtp3	2004	93.5
egy	dtp3	2005	95
…….	….	….	…….

Each row in the table corresponds to an atomic sentence in logic. The table name corresponds to the predicate of the sentence, and each column corresponds to an argument of the predicate.

The reported and survey predicates record the basic input from which the estimates of immunization coverage are derived as output. The next section describes how the output is derived by applying domain-specific rules to the input.

The estimate (output) is represented using the predicate and arguments:

wuenic (*country, vaccine, year, coverage*)

This output can also be represented as in table 3:

**Table 3:** WUEN IC.

*Country*	*Vaccine*	*Year*	*Coverage*
egy	dtp3	2004	97
egy	dtp3	2005	96
…			

In developing a logic-based representation, it is necessary to decide on the choice of predicates and arguments. This corresponds to the decision regarding the choice of relations (or tables) in a relational database. Frequently many alternative representations are possible, and similar considerations apply in both cases. For example, an alternative representation is to employ a single predicate:

data (*source, country, vaccine, year, coverage*)

corresponding to a single table, see table 4:

**Table 4:** Data.

*Source*	*Country*	*Vaccine*	*Year*	*Coverage*
reported	egy	dtp3	2004	97
reported	egy	dtp3	2005	96
survey	egy	dtp3	2004	93.5
survey	egy	dtp3	2005	95
wuenic	egy	dtp3	2004	97
wuenic	egy	dtp3	2005	96
…….	…….	….	….	…….

### Rules (or Conditionals)

The estimates are derived from the data using domain-specific rules [7] expressed as logical conditionals. The domain specific rules can be expressed in symbolic form, which facilitates their computer-based implementation but they can also be expressed in informal natural languages (e.g., English, French). For example, the rule that derives the output estimate from the input data when there are both reported data and survey results in the same year and the two data values are within 10% of one another can be expressed informally as the English language rule:

**Figure 1:**
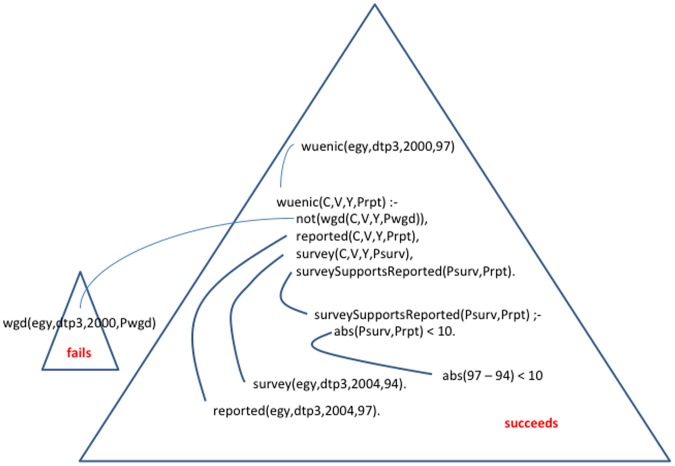
Inference triangle.

If, for a given country/vaccine/year, the reported data are within 10% points of the survey results, then the estimate is the reported data.

As an intermediate representation, between informal English and the symbolic form, the same rule can also be expressed in more precise English:

**For every** *country C, vaccine V, year Y, reported coverage P_rpt_ and survey coverage P_surv_,*

**If** *the coverage in country C, vaccine V, and year Y is reported by*

*the national authorities as P_rpt_*

*and survey coverage result for country C, vaccine V and year Y is P_surv_*

*and the absolute difference between P_surv_ and P_rpt_ is less than 10*

**then** *the estimate for country C, vaccine V and year Y is P_rpt_.*

In symbolic notation of the form of computation logic used in this application the rule above is written in the *conclusion* ***if*** *conditions* form:

Wuenic (*C, V, Y, Prpt*) :-

reported (*C, V, Y, Prpt*),

survey (*C, V, Y, Psurv*),

abs (*Psurv - Prpt*) <10.

Here *C,V,Y,Prpt,Psurv* are variables standing for any country, vaccine, year, reported coverage and survey coverage respectively. The variables are said to be universally quantified. In general variables are represented by expressions beginning with an uppercase character, "and" is represented by a comma, and "if" is represented by ":-".

The *conclusion* of a rule (or conditional) is an *atomic expression,* which is like a fact, consisting of a predicate and its arguments, but, unlike a fact, may contain variables. The *conditions* are a conjunction of atomic expressions or negations of atomic expressions which may also contain variables.

A rule containing universally quantified variables stands for all variable-free instances of the rule. For example, the rule above logically implies the variable-free instance.

wuenic(egy, dtp3, 2004, 97) :-

reported(egy, dtp3, 2004, 97),

survey(egy, dtp3, 2004, 93.5),

abs(93.5 - 97) <10.

The inference engine applies the rule to the atomic sentences representing the basic data using a definition of the arithmetic function abs for absolute value and the relation "***<***" or less than, to derive the estimate:

wuenic (egy, dtp3, 2004, 97).

**Figure 2:**
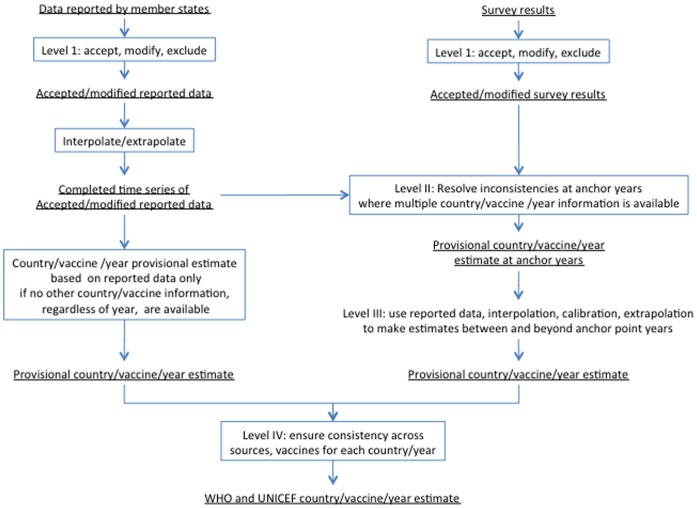
Processing levels.

### Quantitative Computation

Quantitative calculations and procedures can also be implemented in computational logic. For example, an estimate of coverage for the first dose of DTP can be made based on a second degree polynomial function with parameters estimated by a modelled relationship between DTP1 and DTP3 survey results [7].

wuenic (*C,* dtp1, *Y, Pdtp1*) :-

wuenic (*C,* dtp3, *Y, Pdtp3*),

*Pdtp1* is *Pdtp3+* (–0.0066 * (Pdtp3 * Pdtp3))

*(*0.4799 * Pdtp3) +16.67.

Linear interpolation of a value between two other values may be implemented as:

interpolate (*Yearbefore, Pbefore, Yearafter, Pafter, Yearinter, Pinter*) :-

*Pinter* is *Pbefore +*

(Yearinter - Yearbefore) * ((Pafter - Pbefore)/(Yearafter - Yearbefore)).

Interpolation is used, for example, to estimate missing data between two years of reported data.

In both of these examples "is" is an auxiliary predicate representing equality.

### Auxiliary Predicates

In addition to the input predicates, such as reported and survey, calculations, and the output predicate wuenic, our application uses pre-defined functions and predicates, such as "abs" for "absolute value", "*<*" for "less than", "is" for equality. Special-purpose, more abstract auxiliary predicates may be defined and used to express more general rules. For example, the earlier rule:

wuenic (*C, V, Y, Prpt*) :-

reported (*C, V, Y, Prpt*),

survey (*C, V, Y, Psurv*),

abs (*Psurv - Prpt*) <10.

can be represented more generally by replacing the condition abs (*P_surv_ - P_rpt_*) <10 by the abstract condition survey Supports Reported (*P_surv_, P_rpt_* ):

wuenic (*C, V, Y, Prpt*) :-

reported (*C, V, Y, Prpt*),

survey (*C, V, Y, Psurv*),

survey Supports Reported(*Psurv, Prpt*).

The auxiliary predicate used for the abstraction can be defined separately by the rule:

survey Supports Reported (*Psurv, Prpt)* :-

abs (*Psurv - Prpt)* <10.

**Figure 3:**
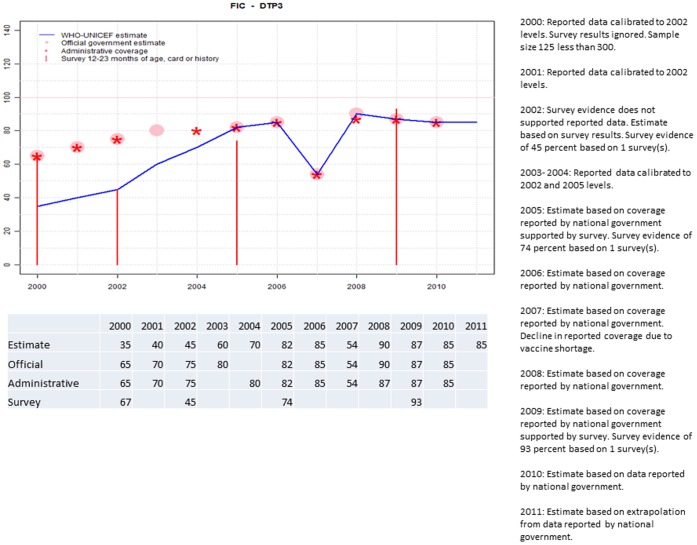
Sample output.

The more general rule using the auxiliary predicate survey Supports Reported is more flexible than the original rule, because it is compatible with other, and more sophisticated, rules for deciding whether survey data supports government reported data. The use of the more general rule facilitates future refinement of the knowledge base by modifying the auxiliary predicate definitions. For example, the definition of the auxiliary predicate survey Supports Reported can be refined to take confidence intervals and other characteristics of the survey into account.

### Negative Conditions

In computation logic, as in relational databases, all information is expressed in terms of positive sentences. Facts are expressed by positive atomic sentences, and rules are expressed by conditionals with positive atomic conclusions. Negative information, expressing that something is not the case, is not represented explicitly, but is assumed to hold implicitly if the corresponding positive information cannot be shown. For example, given only the data:

reported (egy, dtp3, 2006, 97).

it is implicit that:

not (reported(egy, dtp3, 2006, 96)).

Not (reported(egy, dtp3, 2006, 98)).

etc.

Computational logic, unlike conventional symbolic logic, makes use of this assumption that the negation of an atomic sentence holds if the atomic sentence itself does not hold. This assumption is called the *closed world assumption*.

The closed world assumption makes it possible to derive negative conclusions from facts and rules with positive conclusions. This in turn makes it possible to derive further positive conclusions from rules with negative conditions. For example, if the survey does not support the reported data, the conclusion that the estimate is based on the survey results of 85%.

wuenic (egy, dtp3, 2006, 85).

can be derived from the input data:

reported (egy, dtp3, 2006, 97).

survey (egy, dtp3, 2006, 85).

using the additional rule:

wuenic (*C, V, Y, Psurv*) :-

reported (*C, V, Y, Prpt*),

survey (*C, V, Y, Psurv*),

not (survey Supports Reported (*Psurv, Prpt*)).

**Figure 4:**
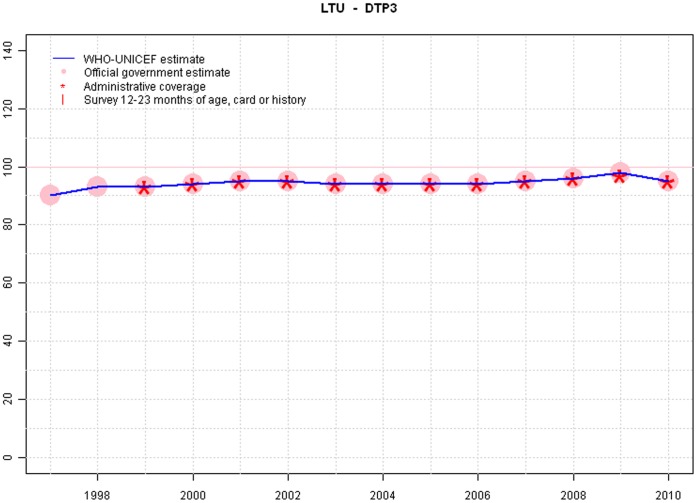
Sample output: Lithuania.

The positive conditions of the rule are satisfied by the input data, and the negative condition is satisfied by the closed world assumption.

### Overriding and Refining Rules

In some instances it is important to be able to override the current rules when their application gives unacceptable conclusions. For example, it may be desirable to override the default estimate produced by a general rule, by taking account of “shocks to the system” or exceptional events rather than the default estimate produced by the rules that may “dampen” or ignore such events.

In many cases this functionality can be achieved by representing the exceptions themselves by general rules. It can also be achieved more simply, however, by adding working group decisions (wgd) to the knowledge base. These decisions are expressed as atomic sentences using an auxiliary predicate wgd having arguments:

wgd (*country, vaccine, year, assigned coverage*)

where *assigned coverage* is the working group’s estimate, which overrides the coverage that would otherwise be assigned by the rules.

There are many reasons why the working group may decide to override the application of a rule. For example, if a survey does not support the reported data for a given country, year and vaccine, but the same survey does support the reported data for all other vaccines, then the working group could decide that the estimate should be based on the reported results for that vaccine as well (perhaps there was a known problem in calculating coverage for that specific vaccine). Such a working group decision, to assign a reported coverage of 94% to the DTP3 coverage estimate in Egypt in 2007, would be represented as.

**Figure 5:**
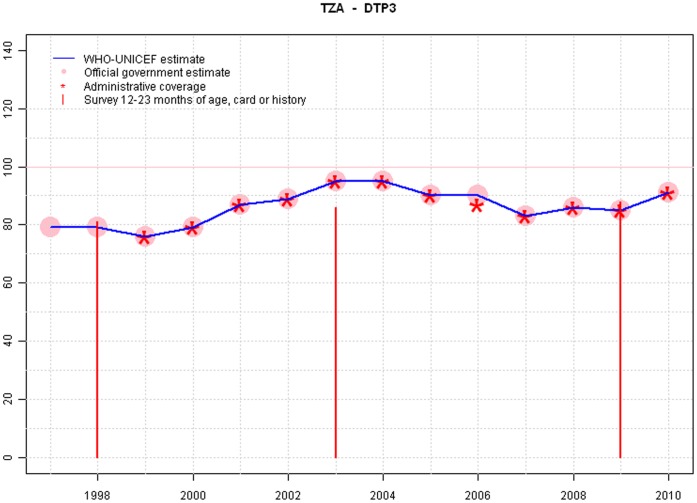
Sample output: Tanzania.

wgd (egy, dtp3, 2007, 94).

To ensure that the rules are overridden by such exceptional decisions, the rules need to include an extra condition, allowing them to engage only if there is no overriding working group decision. For example, the rule for the case where survey does not support the reported data has to be revised to:

wuenic (*C, V, Y, Psurv*) :-

not (wgd(*C, V, Y, Pwgd*)),

reported (*C, V, Y, Prpt*),

survey (*C, V, Y, Psurv*),

not (surveySupportsReported (*Prpt, Psurv*)).

An additional rule needs to be added to assign the estimate by means of the working group decision:

wuenic (*C, V, Y, Pwgd*) :-

wgd (*C, V, Y, Pwgd*).

If working group decisions can be generalized, these generalized exceptions can be implemented as new rules and included in the knowledge base. The most obvious and direct way to refine a knowledge base is simply to amend a definition of a predicate, replacing it by a more sophisticated definition of the same predicate. However, the representation of knowledge as rules also facilitates refinement by adding new rules and by adding new conditions to existing rules. The addition of rules for a given predicate extends the rules to cover more cases, whereas the addition of conditions restricts the rules and prevents them from deriving unsatisfactory conclusions.

**Figure 6:**
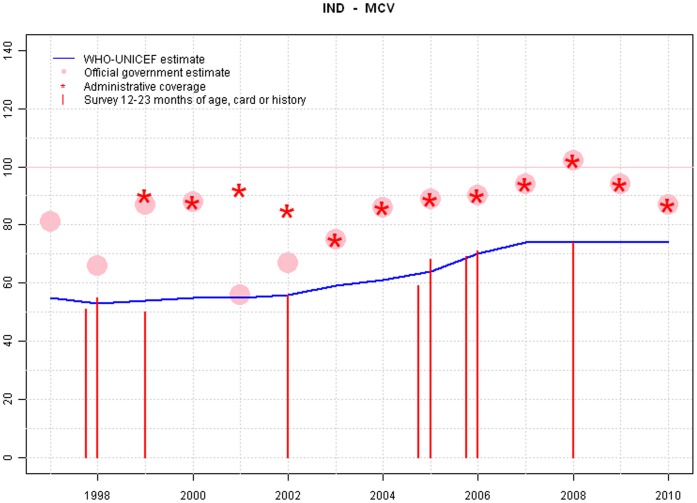
Sample output: India.

### Reasoning

Much of the power of the computational logic lies in the use of inference rules which derive logical consequences of information in the knowledge base. These derivations can be viewed in purely logical terms as systematically applying formal rules of logical inference, which are independent of any application domain. In general, the inference rules can be viewed as filling in a triangle, which has the query (or goal) at the apex, atomic data and other information at the base, and domain specific rules in the interior connecting the atoms and the goal. Some inference engines fill in the triangle top-down; others, bottom-up.

In Figure 1, notice that the top-level goal is to find a value of the variable *P*, such that.

wuenic (egy, dtp3, 2004, *P*)

holds for that value.

For example, given the rules and the data:

wuenic (*C, V, Y, Prpt*) :-

not (wgd(*C, V, Y, Pwgd*)),

reported (*C, V, Y, Prpt*),

survey (*C, V, Y, Psurv*),

survey Supports Reported (*Prpt, Psurv)*.

wuenic (*C, V, Y, Psurv*) :-

not (wgd(*C, V, Y, Pwgd*)),

reported (*C, V, Y, Prpt*),

survey *(C, V, Y, Psurv*),

not (survey Supports Reported (*Prpt, Psurv*)).

wuenic (*C, V, Y, Pwgd*) :-

wgd (*C, V, Y, Pwgd*).

Survey Supports Reported (*Prpt, Psurv*) :-

abs (*Psurv - Prpt*) <10.

reported (egy, dtp3, 2004, 97).

survey (egy, dtp3, 2004, 93.5).

The inference rules derive the value P = 97:

wuenic (egy, dtp3, 2004, 97).

Notice that, in symbolic logic, neither the order in which the rules are written, nor the order in which the conditions of rules are written, affects the results.

### Explanations

The domain-specific rules used to fill in an inference triangle, when made explicit to the user, provide an explanation why the conclusion is a logical consequence of the rules and input data. These explanations are a useful feature which helps to justify the result. If the answer is challenged, the explanation helps to focus attention on those rules and data that are relevant to the derivation of the answer.

More expressive explanations can be generated as part of the output, by adding an extra argument to the output predicate, wuenic. For example,

wuenic (*C, V, Y, P1*, “Reported coverage is supported by survey”) :-

not wgd (*C, V, Y, Pwgd,Explanation*),

reported (*C, V, Y, Prpt*),

survey (*C, V, Y, Psurv*),

survey Supports Reported (*Prpt, Psurv*).

The explanation argument is also added to the wgd predicate, to justify working group decisions. For example:

wgd (egy, dtp3, 2007, 0.94, “While the reported coverage seems to be supported by survey results, the same survey does not support the reported coverage for other vaccines. The estimate is based on the survey results”).

These explanations can be propagated from the working group decisions to the output predicate, using the rule:

wuenic (*C, V, Y, P, Explantation*) :-

wgd (*C, V, Y, P, Explanation*).

For consistency, if an extra argument is added to a predicate in one place, then it must be added to all occurrences of the same predicate. The detailed treatment of explanations in beyond the scope of this paper, and depends in part on the facilities provided by the implementation language. The implementation of explanations in Prolog, for example, is discussed in detail in Bratko [11].

### Further Refinement

In our application, the estimation rules are under constant revision and refinement. For example, at the time of writing, the simple rule, which in its earlier incarnation had the form:

wuenic (*C, V, Y, Prpt*) :-

not wgd (*C, V, Y, Pwg*),

reported (*C, V, Y, Prpt*),

survey (*C, V, Y, Psurv*),

survey Supports Reported (*Prpt, Psurv)*.

has now been replaced by the rule:

wuenic (*C, V, Y, Prpt*, "AP:R", “Reported coverage is supported by survey”) :-

estimate Required (*C, V, Y*),

not wgd (*C, V, Y, Pwgd, Action*),

data (reported, *C, V, Y, Prpt*),

survey (*C, V, Y, Survey Description, Psurv*),

survey Supports Reported (*Prpt, Psurv)*.

Here the additional arguments of the wuenic predicate is the name of the rule used to produce the estimate and the explanation described above. The name of this rule is AP:R (for *anchor point*, resolved to *reported* data), for reasons that are explained in the next section.

An additional predicate, estimate Required (*C, V, Y*), is used to specify the country/vaccine/year combinations for which an estimate should be produced. For example, the following facts state that DTP3 estimates should be produced for Egypt for 2004 and 2005 is represented as:

estimate Required (egy, dtp3, 2004).

estimate Required (egy, dtp3, 2005).

The wgd predicates have been expanded to include a more general *Action*argument, which specifies, in addition to the direct assignment of a coverage estimate, other decisions to override the application of the rules. Other decisions include ignoring data for reasons other than those specified in the current rule set, accepting data ignored by the rule set, and adding comments to provide additional explanations.

The reported predicate has been replaced by a more general data predicate. Other possible values of the first argument are admin (for data based on administrative records reported by national authorities) and gov (for national authorities’ estimate of immunization coverage). These values have proved to be useful for other purposes.

A *Survey Description* argument has been added to the survey predicate which includes detailed information about the survey, including its title, survey type (e.g., Demographic and Health Surveys, the UNICEF Multiple Indicator Surveys, and the Expanded Programme on Immunization cluster surveys), year data collected, percent of immunization cards seen, method used to confirm vaccination (e.g., cards, caretaker report, either), age cohort, and sample size.

Some items of the *Survey Description* argument (e.g., survey title, year of data collection, percent cards seen and sample size) are repeated for each vaccine. A more appropriate representation is to use one predicate having a unique survey identifier and the common items as arguments and a second predicate with the unique survey identifier and coverage specific details as arguments.

### Description of the Estimation System

WHO and UNICEF have used the constructs above to formally describe the rules used to derive immunization coverage estimates. Rules are structured in four levels.

Level one: Accept, modify or exclude coverage data reported by national authorities or obtained through surveys.

Level two: Make estimates at “anchor point” years where there is more than one source of data or information (e.g., data reported by national authorities and survey results). If data are available from only a single source for the entire time series, estimates are made based on these data.

Level three: Make estimates at years between and beyond the anchor point years, completing the time series.

Level four: Compare estimates for consistency and reconcile discrepancies.

Figure 2 illustrates the process.

Level one, the acceptance, modification or exclusion of underlying empirical data is fairly universal in estimation processes. In some instances data may require modification to be comparable with data from other sources. In other instances, data may be excluded from further analysis because they are non-representative of the target population/situation, the collection or analysis of the data may be flawed or they may be incommensurate with the larger body of evidence. Level two is also generally applicable when multiple data points inform a single estimate and combining or resolving differences is necessary. The statistical literature is fairly extensive in this area and include methods from simple averaging to fixed or random effect means to more complex methods; for example, combining data for a more robust estimate is a central motivation in Bayesian analysis. Level three processing is necessary in generating a consistent time series and can be thought of more generally as projecting or predicting measures based on other values. Simple regression models or time-series methods are probably the most common examples. Level four, may not be explicit in all estimation processes but in the case of WUENIC, the comparison of generated values to ensure internal consistency is an important check in preventing the system from producing “nonsensical” results and leading to further refinement of the rules.

### Level One

Each reported and survey data point is passed through a series of “filters” and either a) accepted, b) modified, or c) excluded from further analysis. Two example filters for reported data are:

1. Reported coverage figures ≥100% are excluded from further analysis. While such reports are theoretically possible they are more likely the result of a calculation error, an inaccurate denominator, or an inaccurate estimate of the number of children immunized (numerator).
2. While general trends in immunization coverage are frequently observed, it is rare that large changes occur from one year to the next. Such large changes are more likely to be the result of calculation error, missing reports, or the inclusion of children vaccinated during non-routine, supplemental immunization activities. Large jumps in the level of reported data are excluded unless the working group has reasons to believe that the deviation is due to a genuine change in service delivery.

To facilitate comparison of reported data with survey results and other information, a complete time series is constructed based solely on reported data for all country/vaccine/year combinations for which estimates are required. Its values are the reported data that passed the exclusion filters for any given year. If there are years between two accepted points for which there is no accepted value, the time series value for that year is estimated using linear interpolation. Simple “nearest neighbour” extrapolation (using the value closest in time for that country/vaccine) is used to estimate missing data from the earliest reported data to the beginning of the time series and from the latest reported data forward to the end of the time series.

Survey results are also accepted, modified, or excluded for further analysis. Surveys with sample sizes <300 or with results outside the appropriate age cohort are excluded unless the working group has other reasons to accept the results. If adequate data are available, results for multi-dose antigens (e.g., DTP3, Pol3, etc) are modified for recall bias.

Additional conditions (e.g., inappropriate age cohort, working group decision if survey results are compromised by design or implementation issues) may also lead to surveys being excluded. Working group decisions may override rules that would exclude data points for both reported and survey data. For example, while a survey with a sample size of 299 would be excluded by one of the processing rules, the working group can decide to reinstate the survey and accept the results.

### Level Two

In cases where the only source of data for a country are reports from national authorities, and the working group has no reason to exclude these reports, estimates are based on completed time series of accepted reported data.

For a given country and vaccine, if both survey results and data reported by the national authorities are available, estimates are first made at “anchor point” years where there are multiple sources of data. At these points survey results may support reported data or they may be significantly different. If reported data are within 10% points of survey results and there is no working group decision based on other considerations, the estimate is based on reported data for that year. Alternatively, the estimate is based on survey results if the survey does not support the reported data and the difference between the two is greater than 10% points.

### Level Three

Estimates for years between two anchor point years depend on the way in which estimates are resolved at the anchor points. If surveys support reported data at both anchor point years, then the estimates between the anchor point years are set equal to the reported data as accepted or modified in level one. Otherwise, the estimates are the accepted or modified reported data calibrated to the level of the estimates at the anchor point years. Alternatively, the working group may decide to interpolate between the anchor point estimates providing an accompanying justification. Estimates beyond the earliest and latest anchor points are based on the reported time series if survey results support reported data; otherwise the estimates are based on the reported time series calibrated to the level of the survey.

### Level Four

Levels 1 through 3 operate on data for each country and vaccine independently. Estimates *across* vaccines are reconciled in Level 4. For example, in some countries, DTP1 coverage is underreported because it is not considered the “final” of the three dose DTP series recommended in many national schedules. If DTP3 coverage levels are greater than DTP1 levels or no DTP1 results have been reported, DTP1 coverage is estimated based on a second degree polynomial function describing the relationship between DTP3 coverage and the difference between DTP1 and DTP3 (DTP1 coverage - DTP3 coverage), see section 2.3.

## Results

While the data, domain-specific rules and working group decisions together with rules of logical inference can be used to manually infer estimates, the formal description has been implemented for automated production. Presently, data and information (administrative data, estimates made by national authorities, survey results and working group decisions) are maintained in a Microsoft Access [12] production database. Rules are implemented in XSB Prolog [13]. An R [14] script extracts data from the Access data-base and creates a country-specific file of Prolog predicates of the data, information and working group decisions. XSB Prolog executes the rules using the country-specific file of data and information and produces a file of estimates with the supporting data and working group decisions. An R script reads this file and outputs graphs and LaTex [15] source code of a country-specific summary. LaTex is used to produce country-specific Portable Document Format (PDF) [16] formatted reports. Once data and working group decisions have been updated, it takes approximately 30 seconds to produce each country-specific report. Alternative implementations are certainly possible. An example of the final output is illustrated in Figure 3. The output includes: 1) a graph showing the empirical data and the WHO and UNICEF estimates, 2) a table of data and estimate values, and 3) explanations for each estimate. Text for the explanation is generated automatically by the relevant rules and the working group decisions and comments. The example illustrates:

2000: The 2000 estimate is based on the reported data for 2000 calibrated to the level of the 2002 survey because the 2002 survey does not support the reported data. The 2000 survey results are excluded because the sample size was less than 300 children.

2001: The 2001 estimate is based on the reported data for 2001 calibrated to the level of the 2002 survey because the 2002 survey does not support the reported data.

2002: The 2002 estimate is based on survey results which do not support reported data.

2003–2004: The 2003 and 2004 estimates are based on reported data calibrated to the 2002 estimate and 2005 estimate which is based on reported data confirmed by survey results.

2005: The 2005 estimate is based on the reported data confirmed by survey results.

2006: The 2006 estimate is based on reported data because surveys support reported data in 2005 and 2009.

2007: The 2007 estimate is based on reported data. The formalization includes a condition to exclude reported data that show such extreme changes overage time. However, in this example the working group has ascertained that the decline is not a reporting artefact but rather a real decline in coverage due a shortage of vaccine. A working group decision reinstated the reported data point and the justification for “overriding” the rule was included.

2008: : The 2006 estimate is based on reported data because surveys support reported data in 2005 and 2009.

2009: The 2009 estimate is based on reported data because the 2009 survey results support the reported data.

2010: The 2010 estimate is based on reported data because the 2009 survey results supported reported data.

2011: The 2011 estimate is extrapolated from the reported data because there was no reported data for 2011.

Graphs from Lithuania, Tanzania and India are provide in Figures 4,5,and 6.

## Discussion

WHO and UNICEF have formalized rules used to make immunization coverage estimates using a form of computational logic.

The use of computational logic has an advantage over representation using normal relational database systems in that simple facts (e.g., 2004 reported DTP3 coverage Egypt of 97%) as well as rules that allow us to generalize or infer information from the simple facts in our knowledge base can be included. Exceptions may be stated either as facts or as rules in their own right.

Formalizations in computational logic can be fairly easily implemented in a variety of programming languages. As seen above, predicates resemble data structures, and rules are expressed in logic that is easily represented in both Prolog and expert system shells. We believe that it would be interesting to implement our formalization in the database query and programming language SQL [19].

The formalization of an estimation process raises several important issues. One is ensuring that the interaction between rules provides consistent results. For example, a rule set should not generate multiple estimates for a given country/vaccine/year combination and should generate an estimate for each required combination. A comprehensive test suite is essential during the development of such a formal system.

Another is excessive reliance on mechanical procedures in estimate-making. The best articulated rules set will require refinement and ultimately, human judgment to decide difficult cases and recognize exceptions. For this reason, the working group uses the formalized, explicit rules merely to assist in estimation; it does not delegate the estimation process to the system. Rules are applied to particular cases. If a conclusion is unacceptable or if there is disagreement regarding the conclusion, arguments that the rule should not apply in this case are sought. If persuasive arguments are found, then an exception may be made for the specific case or the general rule may be revised.

Finally, formalization requires that concepts such as “supports” or “is consistent with” be operationalized. We have addressed such issues by providing a precise operational definition for such concepts. An alternative approach is to describe such concepts using fuzzy set theory [17]. Haack [18] provides a critique of these two approaches.

The Institute of Health Metrics and Evaluation (IHME) has developed an alternative approach to estimating immunization coverage, using novel statistical techniques [20], which at first glance seems dramatically different from a rule-based system. At an abstract level, however, both approaches have many features in common. Both have a first level at which data are evaluated, a second level at which estimates are made in anchor years where there are survey data, and a third level in which estimates are made for non-anchor years, using a form of calibration. And, at the top-most level the IHME approach could also be formalized within a computation logic framework. In this case the IHME approach would differ from the WHO and UNICEF approach primarily by employing more complex quantitative analysis and paying less attention to context-specific and judgemental matters.

In terms of results, both approaches can be quite similar. For the common set of 3864 estimates produced by Lim et al and WHO and UNICEF, (note that WHO and UNICEF do not produce estimates for non-member states; for example, no estimates were produced for the republics of the former Soviet Union for the period 1986–1991 or for Timor-Leste from 1986 until they gained independence in 2001), 2989 (77%) of the WHO and UNICEF estimates fell within the 95% confidence bounds of the IHME estimates. Differences ranged from −64.5% points (Equatorial Guinea, 1990, IHME  = 12.5%, WHO and UNICEF  = 77%) to 61.9% points (Rwanda, 1994, IHME  = 89.4, WHO and UNICEF  = 23%). The median difference was –5.0% points with an inter-quartile range of −7.1% points to 11% points. In many cases the difference can be attributed to the inclusion/exclusion of particular surveys. For example two surveys conducted in Equatorial Guinea in 1990 and 1993 showing coverage of 77% were accepted by WHO and UNICEF and excluded by IHME. The Albanian 2000 Multiple Indicator Cluster Survey was excluded by WHO and UNICEF because immunization were kept at local health centres, which were not visited, rather than in the child’s home. In other instances the differences can be attributed to incorporation of context-specific information by WHO and UNICEF. For example, WHO and UNICEF accepted a dramatic decline reported in immunization coverage for Rwanda in 1994 associated with the 1994 genocide. The IHME estimates show no impact of this event on immunization coverage.

An advantage of the statistical approach by Lim et. al. is that their model allows the estimation of uncertainty due to variance in their data. While the WHO and UNICEF estimates are based only on country-specific data, the IHME estimates are predicated on a systematic relationship between survey data and reported data across countries, a relationship that is generalized to countries with no surveys. However, the relationship between survey results and reported data is weak and is exposed in the wide uncertainty bounds of IHME estimates for non-survey years and in estimates for countries with no surveys [20], [21].

An alternative to both the IHME and WHO and UNICEF would be a Bayesian approach which would allow an estimate to be based on both prior information and observed data from a variety of sources [22]. The challenge in constructing a Bayesian model is to establish not only the prior probabilities for coverage estimates but also the necessity of specifying the likelihood functions for the observed data. Unlike the WHO and UNICEF approach, both the IHME and a Bayesian approach produce uncertainty ranges.

In summary, the production of official statistics by any number of methods frequently requires expert judgement to evaluate and reconcile data of unknown and varying quality from multiple and potentially conflicting sources. The use of computational logic extends the flexibility of standard statistical approaches by providing the tools for incorporating analyst's judgement, integrating multiple data sources and modelling methods, ensuring transparency and replicability, and making documentation computationally accessible. We believe that formalization using computational logic can be usefully applied to a wide range of official statistics.

## Supporting Information

Annotated Bibliography S1 **Knowledge representation and reasoning using computational logic: an annotated bibliography.**
(DOCX)
