# Supplementary material for: A Formal Representation of the WHO and UNICEF Estimates of National Immunization Coverage: A Computational Logic Approach
Source: PLoS One. 2012 Oct 25;7(10):e47806. doi: 10.1371/journal.pone.0047806 (PMC3485034; doi:10.1371/journal.pone.0047806)
Supplement: Annotated Bibliography S1 — Knowledge representation and reasoning using computational logic: an annotated bibliography. (DOCX) [file pone.0047806.s001.docx]

Annotated Bibliography S1: Knowledge representation and reasoning using computational logic: an annotated bibliography

Kowalski[5] presents a comprehensive, informal introduction to computational logical for a general audience. Brachman and Levesque[22] provide an introduction to knowledge representation and reasoning. Russell and Norvig[23] is a popular university-level text on artificial intelligence and chapters 7 through 9 present material on knowledge representation and reasoning. Sowa[24] provides a readable overview of the field. Davis[25] contains practical advice on representing domain knowledge in formal logic.

Computational logic is an extension of first order predicate logic. Logic has long been used to unambiguously represent knowledge, and computers can efficiently prove theorems expressed in the clausal form of logic. A readable introduction to logic is Bennet[26]. Kowalski[27] presents the use of clausal logic for representing knowledge and general problem solving; Richards is a gentle introduction to clausal form logic[28]. Barwise and Etchemendy[29] is a classroom text with a computer science approach and includes a CD-ROM of interactive exercises. Enderton[30] provides a more advanced approach to logic. Genesereth and Nilisson[31] is a classic text on the application and foundations of logic in artificial intelligence.
